# Molecular characterization, expression analysis and heterologous expression of two translationally controlled tumor protein genes from *Cucumis sativus*

**DOI:** 10.1371/journal.pone.0184872

**Published:** 2017-09-19

**Authors:** Xiang nan Meng, Qiu min Chen, Hai yan Fan, Tie feng Song, Na Cui, Ju yong Zhao, Shu min Jia, Ke xin Meng

**Affiliations:** 1 College of Horticulture, Shenyang Agricultural University, Shenyang, People’s Republic of China; 2 College of Bioscience and Biotechnology, Shenyang Agricultural University, Shenyang, People’s Republic of China; 3 Key Laboratory of Protected Horticulture of Ministry of Education, Shenyang Agricultural University, Shenyang, People’s Republic of China; 4 Liaoning Academy of Agricultural Sciences, Shenyang, People’s Republic of China; 5 Foreign Languages Department, Shenyang Agricultural University, Shenyang, People’s Republic of China; Huazhong University of Science and Technology, CHINA

## Abstract

The translationally controlled tumor protein (TCTP) is a family of abundant and ubiquitous proteins involved in several important primary functions. Cucumbers harbor two *TCTP* genes, *CsTCTP1* and *CsTCTP2*; however, their functional roles remain unclear. In this study, we isolated *CsTCTP1* and *CsTCTP2* (XP-004134215 and XP-004135602, respectively) promoters, full-length cDNA and genomic sequences from *Cucumis sativus*. Bioinformatics analysis, containing *cis*-acting elements, structural domains, phylogenetic tree and conserved motifs, suggested the conservation and divergence of CsTCTP1 and CsTCTP2, thus providing knowledge regarding their functions. Expression analysis indicated that *CsTCTP1* and *CsTCTP2* exhibited tissue-specific expression and were regulated by biotic or abiotic stresses in *C*. *sativus*. Furthermore, *CsTCTP1* and *CsTCTP2* were regulated by ABA and may be associated with the TOR (target of rapamycin) signaling pathway. In a prokaryotic expression analysis, *CsTCTP1* and *CsTCTP2* showed positive responses to salt and heat stresses and a negative response to drought and HgCl_2_ stresses. TCTP may exert multiple functions in various cellular processes.

## Introduction

The translationally controlled tumor protein (TCTP) is a highly conserved protein in eukaryotic phyla. It was initially discovered in Ehrlich ascites tumor cells and has subsequently been studied in animals [1, 2]. Extant data suggest that TCTP exerts multiple functions in various cellular processes such as cell growth, differentiation, organ size, apoptosis, signaling pathway, stimulus and immune responses [3–6]. TCTP functions as a GEF (guanine nucleotide exchange factor) of Ras GTPase Rheb in *Drosophila melanogaster* and is related to the TOR (target of rapamycin) signaling pathway [7]. The high conservation and ubiquitous presence of this protein underscores its similar mechanisms among plant and nonplant homologs. The notion that its role is similar was first supported by the fact that the *Arabidopsis TCTP* gene can rescue the corresponding *Drosophila TCTP* mutant [8, 9].

The first report of plant *TCTP* was obtained from *Medicago sativa* [10]. Several *TCTP* genes have been identified from different plant species by comparative transcriptomic and proteomic studies [11, 12]. Furthermore, *AtTCTP* overexpression enhanced drought tolerance in *Arabidopsis* and played a role in ABA-mediated stomatal movement [13]. RNAi of *Rpf41* (a homolog of TCTP) in *Robinia pseudoacaia* decreased the root and stem length, fresh weight and nodule number per plant [14]. To date, work on *TCTP* in plants has focused mostly on *Arabidopsis*; however, few experiments have been conducted. The Arabidopsis genome consists of two TCTP sequences, AtTCTP1 (NP-188286) and AtTCTP2 (NP-187205). The molecular function of the first sequence is better studied. The latter sequence was once thought to be a pseudogene; however, AtTCTP2 appears to play crucial roles in plant regeneration according to Toscano-Morales [15]. These published data suggest a role of AtTCTP1 in the regulation of growth, the control of programmed cell death and the response to stress signals [9, 16]. Silencing of the *AtTCTP1* gene in *Arabidopsis* caused slow vegetative growth, leaf expansion and lateral root formation [17]. AtTCTP2 is essential for viability and enhances plant regeneration. Indeed, silencing of the *AtTCTP2* gene in *Arabidopsis* displays a lethal phenotype [18]. The molecular function and physiological mechanism of TCTP in plants are still not fully understood; elucidating them is of immense importance.

Through a 2-D gel analysis, TCTP (XP-004134215) was found to be involved in the response of cucumber to *Sphaerotheca fuliginea* at the protein level [12]. However, the functional characterization of CsTCTPs remains unclear. Cucumber harbors 2 *TCTP* genes. Here, we report a detailed expression analysis and molecular characterization of CsTCTP1 and CsTCTP2 (XP-004134215 and XP-004135602, respectively). The expression patterns of *CsTCTP1* and *CsTCTP2* were determined in different tissues and under various treatments. In addition, *E*. *coli* BL21 strains overexpressing *CsTCTP1* and *CsTCTP2* exhibited varying degrees of tolerance to heat, salt, drought and mercury stress. The study provides new insight into TCTP in cucumber and will be helpful in further improving cucumber performance under stresses.

## Materials and methods

### Plant materials and treatments

Two cucumber sister lines, one that is highly resistant (B21-a-2-1-2) and one that is highly susceptible (B21-a-2-2-2) to *S*. *fuliginea*, were obtained from Liaoning Academy of Agricultural Sciences. These lines were selected from a segregated population derived from fourth generation selfing of a cultivar from South Korea. The two lines are similar in terms of plant type, commodity characteristics, resistance to Fusarium wilt and downy mildew but differ in their traits of resistance to powdery mildew.

Cucumber seeds of both lines were grown in soil with a 16 h photoperiod at 25°C/18°C (day/night). For biotic stress, at 3^rd^-4^th^ leaf stage, the second leaf blades of B21-a-2-1-2 and B21-a-2-2-2 were inoculated with *S*. *fuliginea* by gently rubbing mildewed leaves. For abiotic stress and stimuli treatments, 3^rd^-4^th^ leaf stage seedlings of B21-a-2-1-2 were treated with 45°C (for heat stress) or 4°C (for cold stress), irrigated with water containing 20% PEG (m/v, for drought stress), or sprayed with water containing 100 mM NaCl (for salinity stress), 10 mM CaCl_2_, 10 μM H_2_O_2_, 100 μM ABA, 100 μM MeJA, 1 mM SA or 1% (v/v) Ethrel. During the treatments, six-time points (0, 12, 24, 48, 72, and 144 h) under biotic stress, three-time points (12, 24, and 48 h) under stimuli treatments, and five-time points (0, 3, 6, 12, and 24 h) under abiotic stresses. Then, the second leaves collected at each time point were placed in liquid nitrogen and stored at -80°C until further experiments.

### Extraction of genomic DNA, total RNA and cDNA synthesis

Genomic DNA was isolated from the two cucumber sister lines B21-a-2-1-2 and B21-a-2-2-2 seedling leaves with a Plant Genomic DNA kit (TianGen Biotech, China) according to the manufacturer’s instructions. Total RNA from various samples was isolated using RNAprep Pure Plant Kit, and cDNA was generated using a FastQuant cDNA first strand synthesis kit (TianGen Biotech, China) according to the manufacturer’s instructions. The DNA and RNA were detected on 1.2% agarose gels, and the purity of the DNA and RNA was determined by spectrophotometry (Biodrop).

### Cloning of CsTCTP1 and CsTCTP2 promoter, DNA and cDNA sequences

The nucleotide sequence of *Cucumis sativus* L. *TCTPs* (*CsTCTP1*, accession: XP-004134215 and *CsTCTP2*, accession: XP-004135602) was retrieved from the NCBI database (http://www.ncbi.nlm.nih.gov/). Amplifications of *CsTCTP1* and *CsTCTP2* cDNAs were performed with primers 1- cDNA F/1- cDNA R and 2- cDNA F/2- cDNA R on first-strand cDNA templates of B21-a-2-1-2 and B21-a-2-2-2 in a Mastercycler (BIO-RAD) under the following conditions: 94°C for 5 min, 94°C for 30 s, 58°C for 30 s, 72°C for 1 min, 32 cycles, and 72°C for 10 min. The PCR products were gel-purified, cloned into pMD18-T vector (TaKaRa, China) and sequenced (Sangon Biotech, China).

Genomic PCRs were performed with primers 1- DNA F/1- DNA R and 2- DNA F/2- DNA R on DNA templates of B21-a-2-1-2 and B21-a-2-2-2 as follows: 94°C for 5 min, 94°C for 30 s, 62°C for 30 s, 72°C for 2 min, 36 cycles, and 72°C for 10 min. For promoter analysis, the promoter regions of *CsTCTP1* and *CsTCTP2* (approximately -2.0 kb upsteam of translation initiation site) were also isolated from B21-a-2-1-2 and B21-a-2-2-2 genomic DNA based on 1- P F/1- P R primers and 2- P F/2- P R primers. PCR conditions used were as follows: 94°C for 5 min, 94°C for 30 s, 60°C for 30 s, 72°C for 2 min, 38 cycles, and 72°C for 10 min. The PCR products were gel purified, cloned and sequenced.

### qRT-PCR analysis

Quantitative real-time PCR was conducted using the SYBR Green I 96-I system (Roche fluorescence quantitative PCR instrument, Basle). Reaction mixtures consisted of 4.5 μL of 2×SuperReal PreMix Plus (TianGen Biotech, China), a mixture of primers (0.2 μL of forward and reverse primer for proper gene), 4.3 μL of RNase-Free ddH_2_O and 1 μL of cDNA. The PCR program was set up in seven stages: (1) 95°C for 15 min (pre-incubation), (2) 95°C for 10 s, (3) 58°C for 20 s, (4) 72°C for 30 s, (3) repeated 40 times (amplification), (5) 95°C for 0.5 s, (6) 60°C for 1 min and (melt) (7) 50°C for 30 s (cooling). The primers were synthesized by BGI Tech (China), and the PCR reaction quality was estimated based on melting curves. *18s rRNA* was used as an internal control for determining transcript levels in cucumber. The gene-specific primers employed are shown in S1 Table. Two independent biological replicates and three technical replicates for each biological replicate were run, and the significance was determined by *t*-test using SPSS statistical software (P < 0.05).

### *CsTCTP1* and *CsTCTP2* overexpression in bacteria

The insert fragment was digested with *Kpn* I+ *Sac* I from pMD18-T vector and then ligated into the expression vector pET30a (+) digested with the same enzymes, resulting in constructed vectors (pET30a-*CsTCTP1* or pET30a-*CsTCTP2*). The constructed vectors and empty vector (pET30a) were subsequently transformed into *E*. *coli* BL21 (DE3). The transformed cells were inoculated into LB containing 50μg/mL kanamycin at 28°C, 170 rpm until OD_600_ reached 0.6.

IPTG (final concentration to 1 mM) induced prokaryotic expression products for 0 h, 3 h, 6 h and 12 h at 28°C. Meanwhile, BL21 + pET30a (control) was induced in 1 mM IPTG for 0 h and 12 h. The cells from 1 mL of culture were harvested by centrifuging at 10 000 rpm for 10 min at 4°C, dissolved in 20 μL 2 × SDS loading buffer and boiled for 5 min. All samples were analyzed by SDS-PAGE.

For assays of tolerance to various stresses, bacterial cells containing constructed vectors and the empty vector were induced in 1 mM IPTG for 12 h at 28°C. The cells were diluted to 0.6 (OD_600_) and further diluted to 10−^3^ and 10−^4^. Then, 10 μL of 10−^3^ and 10−^4^ diluted cells was spotted on LB plates containing 50 μg/mL kanamycin at 37°C, 45°C and 55°C (for heat stress) or on LB plates with NaCl (250 mM, 500 mM and 750 mM, for salt stress), mannitol (0.4 M, 0.8 M and 1.2 M, for drought stress), or HgCl_2_ (15 μM, 25 μM and 35 μM, for mercury stress) at 37°C overnight. The cells were also added to LB containing 50 μg/mL kanamycin at 45°C or to LB liquid medium adding 500 mM NaCl, 0.8 M mannitol or 25 μM HgCl_2_ at 37°C, 200 rpm. Cell growth was measured every 2 h by OD_600_ [19–21]. Assays of response to stresses were repeated at least three times. Each treatment was performed at least twice.

### Statistical and bioinformatics analysis

Primer design and sequence alignment were conducted in DNAman. Promoter analysis was performed using BDGP, PLACE (www.dna.affrc.go.jp) and Plant CARE (http://bioinformatics.psb.ugent.be/webtools/plantcare/html). The phylogenetic tree was conducted in MEGA 6.0. The sequence was analyzed with MEME software (http://meme-suite.org/tools/meme), ProtParam Tool (http://web.expasy.org/protparam/), Plant-mPLoc (http://www.csbio.sjtu.edu.cn/bioinf/plant-multi/), SMART (http://smart.embl-heidelberg.de/) and InterProScan (http://www.ebi.ac.uk/Tools/pfa/iprscan/).

## Results

### Isolation and characterization of CsTCTP1 and CsTCTP2

The cucumber genome harbors two *TCTP* genes. Here, we termed the gene that shows high similarity to *AtTCTP1* as *CsTCTP1* (accession: XP-004134215) and the other gene as *CsTCTP2* (accession: XP-004135602). Using the RT-PCR method, the promoters, cDNA and DNA sequences of *CsTCTP1* and *CsTCTP2* were amplified, cloned and sequenced. Subsequently, we compared the sequences of the two sister cucumber lines (B21-a-2-2-2 and B21-a-2-1-2) obtained here with other cucumber TCTP sequences available in the NCBI databases and found no differences between them.

Using 1/2-P F and 1/2-P R primers on cucumber genomic DNA, we obtained the *CsTCTP1* promoter sequence that contains 2096 bp upstream of the translation start site and has an AT content of 74% and the *CsTCTP2* the promoter sequence that contains 2015 bp upstream and has an AT content of 74%. The two promoter sequences shared 45% nucleotide identity, suggesting that *CsTCTP1* and *CsTCTP2* may exhibit different expression profiles. The transcriptional start sites (TSS) of *CsTCTP1* and *CsTCTP2* were found 87 bp and 67 bp upstream from the translation start site, respectively. The putative TATA-box was found at position -28 with respect to the TSS. Putative *cis*-regulatory elements were also deciphered in the *CsTCTP1* and *CsTCTP2* promoter sequences using Plant CARE and PLACE databases; the results are summarized in S1 and S2 Tables. We identified ABRE, TC-rich repeats and circadian in both cucumber promoters, which are important *cis*-acting elements involved in abscisic acid responsiveness, defense and stress responsiveness and circadian control. Numerous *cis*-elements associated with light responsiveness, such as ACE, TCT-motif and G-box, were also found in both promoters. TCA-element, HSE, and MBS were identified only in the *CsTCTP1* promoter sequence. TCA-element, HSE, and MBS are involved in responsiveness to salicylic acid, heat and drought responsiveness, respectively. Two structure-related *cis*-acting elements known as 3-AF3 binding site and Box III were found at position 618 (+) and 773 (+) only in the *CsTCTP2* promoter. Searching the *CsTCTP2* promoter also resulted in the discovery of several *cis*-elements, including ethylene, zein metabolism regulation, anaerobic induction, endosperm expression, fungal elicitor and shoot-specific responsive elements.

A genomic DNA sequence containing a 2211 bp coding region of the *CsTCTP1* gene was obtained by amplification with 1-DNA F and 1-DNA R primers, and a 1679 bp coding region of the *CsTCTP2* gene was obtained by amplification with 2-DNA F and 2-DNA R primers as described in the “Materials and methods” section. Our results showed that the *CsTCTP1* coding region is composed of four introns (i1, i2, i3 and i4 with 578 bp, 181 bp, 84 bp and 496 bp, respectively) and five extrons (e1, e2, e3, e4 and e5 with 28 bp, 74 bp, 129 bp, 158 bp and 118 bp, respectively); *CsTCTP2* coding region is constituted by four introns (i1, i2, i3 and i4 with 509 bp, 113 bp, 89 bp and 112 bp, respectively) and five extrons (e1, e2, e3, e4 and e5 with 28 bp, 74 bp, 129 bp, 158 bp and 118 bp, respectively), which matches the cDNA sequence. The full-length cDNA of *CsTCTP1* was 872 bp in size with a predicted 507 bp open reading frame (ORF), 147 bp 5’-UTR and 218 bp 3’-UTR; the full-length cDNA of *CsTCTP2* was 856 bp in size with a predicted 507 bp ORF, 63 bp 5’-UTR and 286 bp 3’-UTR.

**Fig 1:**
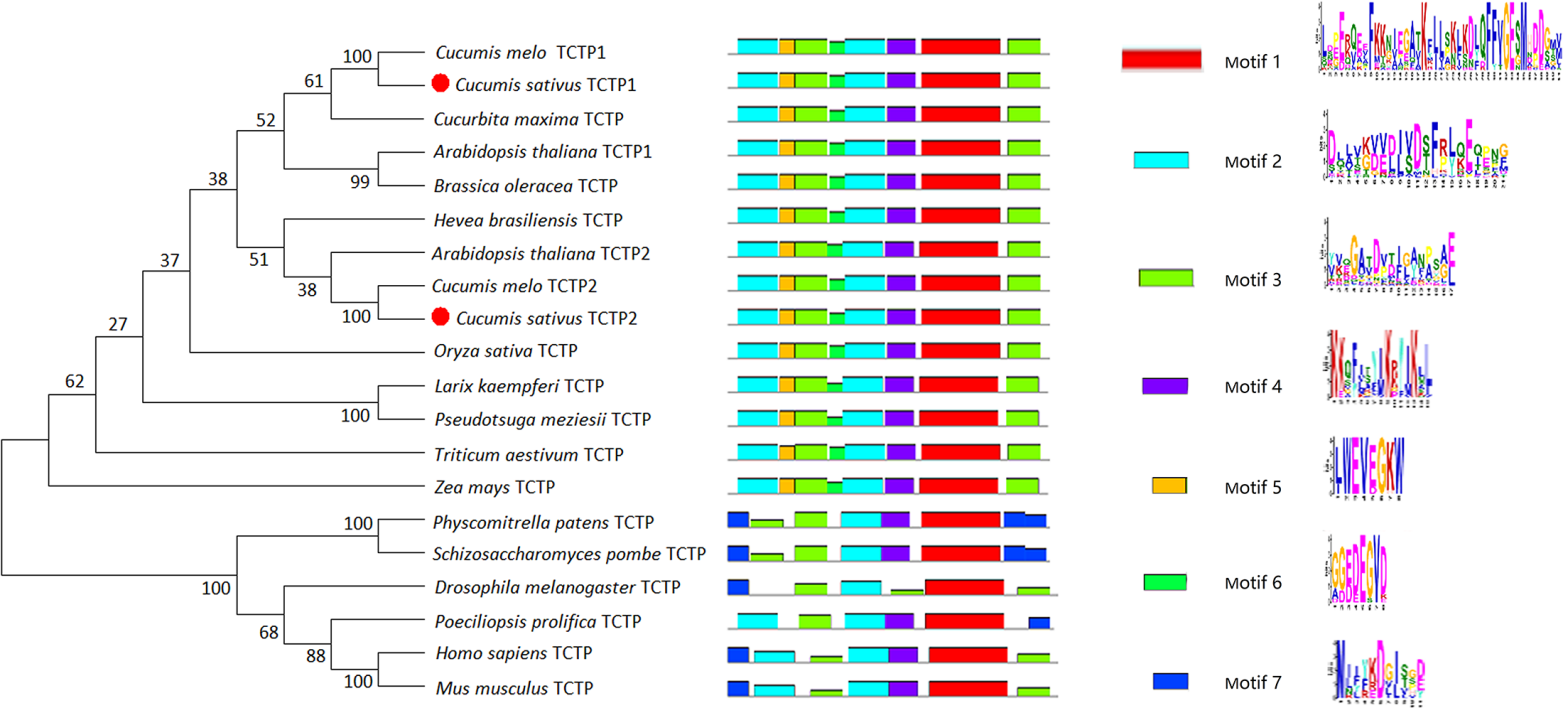
Phylogenetic and conserved protein motifs of TCTPs. The phylogenetic tree of TCTP protein sequences included *Homo sapiens* TCTP (P13693), *Mus musculus* TCTP (P63028), *Drosophila melanogaster* TCTP (Q9VGS2), *Poeciliopsis prolifica* TCTP (JA088771), *Cucumis sativus* TCTP1 (XP-004134215), *Cucumis sativus* TCTP2 (XP-004135602), *Cucurbita maxima* TCTP (ABC02401), *Cucumis melo* TCTP1 (AAF40198), *Cucumis melo* TCTP2 (XP_008450611), *Arabidopsis thaliana* TCTP 1 (NP-188286), *Arabidopsis thaliana* TCTP 2 (NP-187205), *Zea mays* TCTP (Q8H6A5), *Triticum aestivum* TCTP (Q8LRM8), *Oryza sativa* TCTP (XP_015617060), *Hevea brasiliensis* TCTP (Q9ZSW9), *Brassica oleracea* TCTP (Q944W6), *Larix kaempferi* TCTP (AGW01241), *Pseudotsuga meziesii* TCTP (Q9ZRX0), *Schizosaccharomyces pombe* TCTP (Q10344), and *Physcomitrella patens* TCTP (Q10344). The construction of the tree was conducted with Mega 6.0. Numbers above the branches indicate bootstrap values.

cDNA sequence analysis showed that *CsTCTP1* encodes a polypeptide of 168 amino acids with a predicted molecular weight of 18 kDa and a pI of 4.56; *CsTCTP2* encodes a polypeptide of 168 amino acids with a predicted molecular weight of 19 kDa and a pI of 4.35. Sequence comparison of CsTCTP1 and CsTCTP2 showed 77% identity at the amino acid level and 73% at the nucleotide level. Phylogenetic tree analysis showed that CsTCTP1 was closely related to *Cucumis melo* TCTP; CsTCTP2 was closely related to *Arabidopsis thaliana* TCTP2. Conserved motif analysis showed that five types of motifs were common among all eukaryotic phyla analyzed here and that all TCTPs had a highly conservative motif 1 and motif 4 at N-terminal (Fig 1). Thus, the function of different *TCTP* genes may be similar but do not overlap completely.

**Fig 2:**
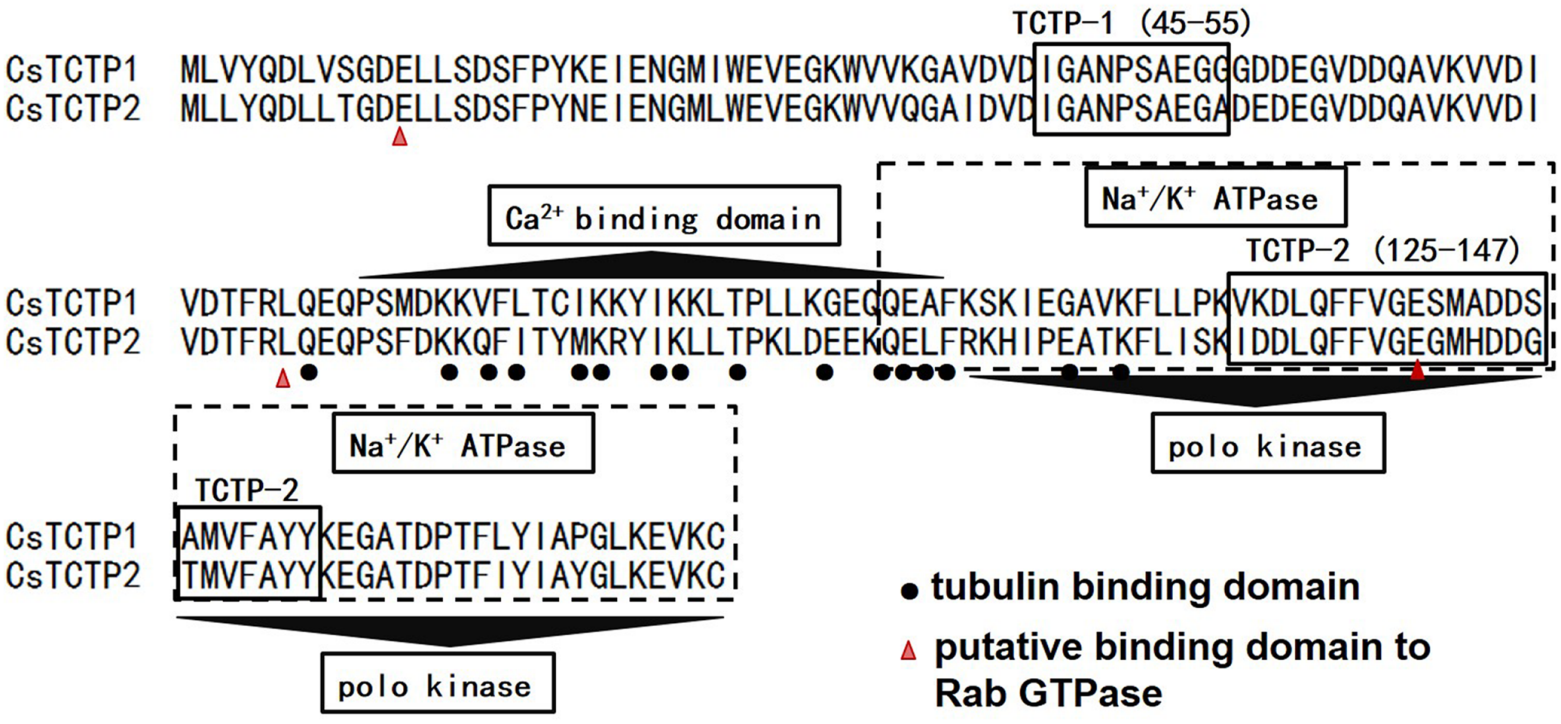
Putative structural domains of CsTCTP1 and CsTCTP2.

Based on data analysis in SMART and InterProScan, CsTCTP1 and CsTCTP2 have several typical TCTP domains, such as a Ca^2+^ binding domain (80–110), cell cycle-controlling polo kinase (111–168), Na^+^/K^+^ ATPase (107–168), tubulin binding domain, putative binding domain to Rab GTPase, TCTP1 (45–55) and TCTP2 (125–147) (Fig 2). Our results suggested that CsTCTP1 and CsTCTP2 are both typical TCTP proteins and that TCTP might have a specialized function in cucumber plants.

### Expression analysis of *CsTCTP1* and *CsTCTP2* in cucumber

#### Expression patterns of *CsTCTP1* and *CsTCTP2*

**Fig 3:**
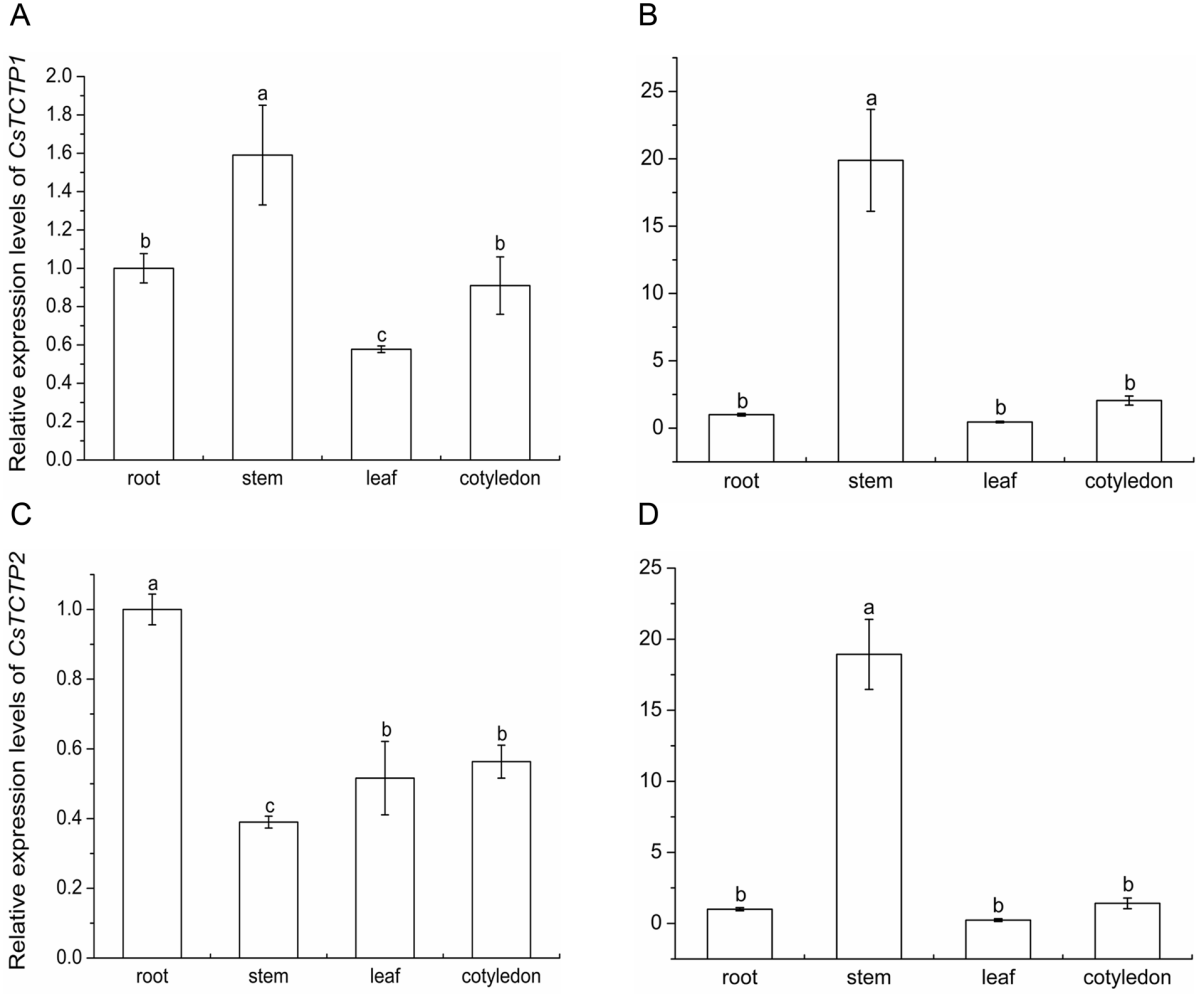
Relative expression levels of *CsTCTP1* in various tissues of the susceptible variety (a) and resistant variety (b). Relative expression levels of *CsTCTP2* in various tissues of the susceptible variety (c) and resistant variety (d). The expression level in the root was normalized as 1. Data represent means ± SE of three biological replicates. Letters indicate significant differences at P < 0.05 compared with the root by Student’s *t*-test.

CsTCTP1 was found to be differentially expressed in both highly resistant and highly susceptible cucumber leaves under *S*. *fuliginea* stress in our previous study. To gain insight into *CsTCTP1* and *CsTCTP2* function, we detected their expression patterns in various tissues by qRT-PCR. Consistent with previous studies [22, 23], *CsTCTP1* and *CsTCTP2* were expressed in all examined tissues (roots, stems, leaves and cotyledons). Among these tissues, *CsTCTP1* was highly expressed in the stems of both sister cucumber lines B21-a-2-1-2 and B21-a-2-2-2, while *CsTCTP2* was highly expressed in the stems of B21-a-2-1-2 and in the roots of B21-a-2-2-2 (Fig 3). These results demonstrated that *CsTCTP1* and *CsTCTP2* are both ubiquitously expressed in all analyzed plant tissues and exhibit tissue-specific expression.

#### Expression of *CsTCTP1* and *CsTCTP2* in response to *S*. *fuliginea*

**Fig 4:**
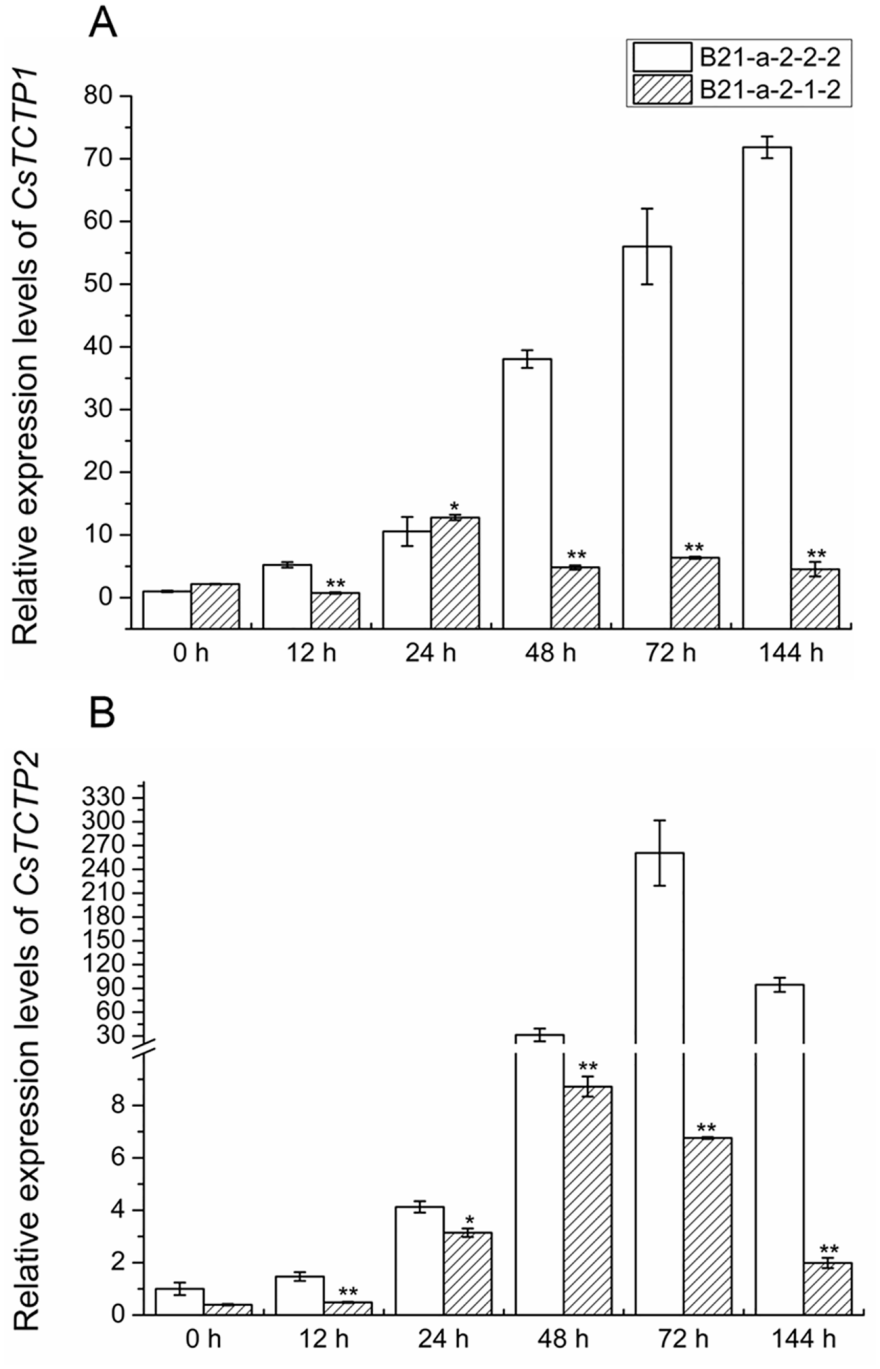
Relative expression levels of *CsTCTP1* and *CsTCTP2* in resistant (B21-a-2-1-2) and susceptible (B21-a-2-2-2) varieties inoculated with *S*. *fuliginea*. The expression level in B21-a-2-2-2 was normalized as 1. Data represent means ± SEs of three biological replicates. Asterisk or asterisks indicate significant differences at P < 0.05 or P < 0.01, respectively, compared with B21-a-2-2-2 by Student’s *t*-test.

TCTP may be regulated by *Erysiphe graminis* (the causal agent of wheat powdery mildew) [24]. Interestingly, we also found that CsTCTP1 seems to be involved in response to *S*. *fuliginea*, the causal agent of powdery mildew in cucumber in previous work. To examine whether *CsTCTP1* and *CsTCTP2* are affected by *S*. *fuliginea*, the expression patterns of *CsTCTP1* and *CsTCTP2* in both highly resistant and highly susceptible cucumber leaves at each corresponding time point were analyzed (Fig 4). During the cucumber-*S*. *fuliginea* interactions, the *CsTCTP1* transcript level increased continuously for 144 h in the susceptible variety B21-a-2-2-2. In the resistant variety B21-a-2-1-2, *CsTCTP1* transcripts reached the highest level at 24 h of *S*. *fuliginea* treatment. *CsTCTP2* appeared to be highly expressed in the susceptible line than that in the resistant line after infestation. Also, *CsTCTP2* transcripts showed similar expression profiles in the two lines. *CsTCTP2* initially increased and then decreased, with the maximum accumulation in the resistant and susceptible lines observed at 48 h and 72 h post inoculation, respectively. It is worth mentioning that the maximum expression of *CsTCTP1* and *CsTCTP2* appeared to be earlier in the resistant variety than in the susceptible variety when inoculated with *S*. *fuliginea*.

#### Expression of *CsTCTP1* and *CsTCTP2* in response to various extracellular stimuli

**Fig 5:**
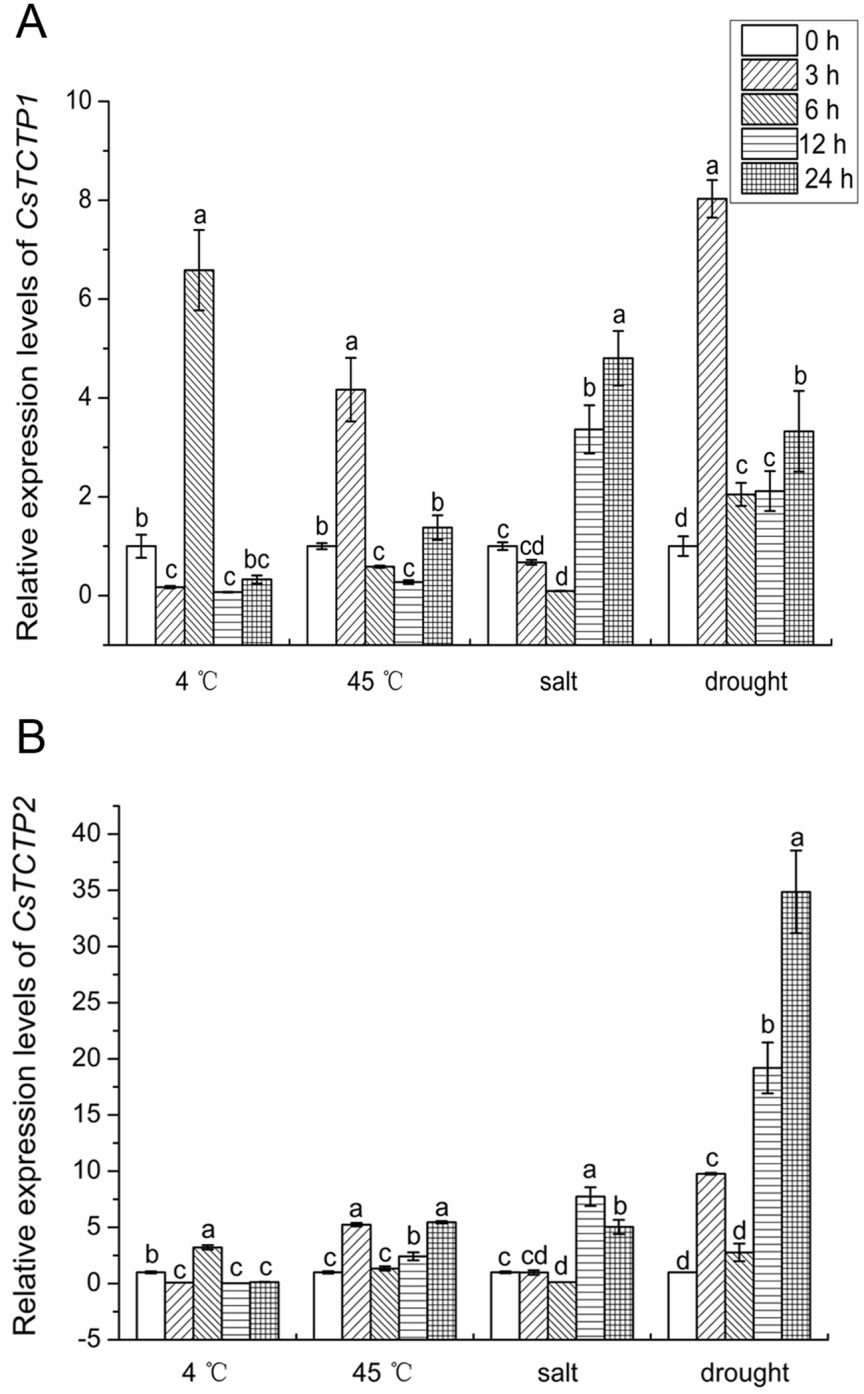
Relative expression levels of *CsTCTP1* and *CsTCTP2* in a resistant (B21-a-2-1-2) variety under various abiotic stresses. The expression level at 0 h was normalized as 1. Data represent means ± SEs of three biological replicates. Letters indicate significant differences at P < 0.05 compared with 0 h treatment by Student’s *t*-test.

Expression of *CsTCTP1* and *CsTCTP2* was also observed under abiotic stresses in the leaves of the resistant variety B21-a-2-1-2 (Fig 5). Under low-temperature and high-temperature stresses, *CsTCTP1* and *CsTCTP2* demonstrated a wave expression pattern and reached the highest level at 6 h and 3 h, respectively. During high salt treatment, *CsTCTP1* and *CsTCTP2* slightly decreased and then increased, with the highest level at 24 h and 12 h, respectively. Under drought treatment, *CsTCTP1* and *CsTCTP2* were constantly up-regulated; the maximum accumulation occurred at 3 h and 24 h, respectively. These results reveal that *CsTCTP1* and *CsTCTP2* might play important roles in a variety of stress responses in cucumber plants.

**Fig 6:**
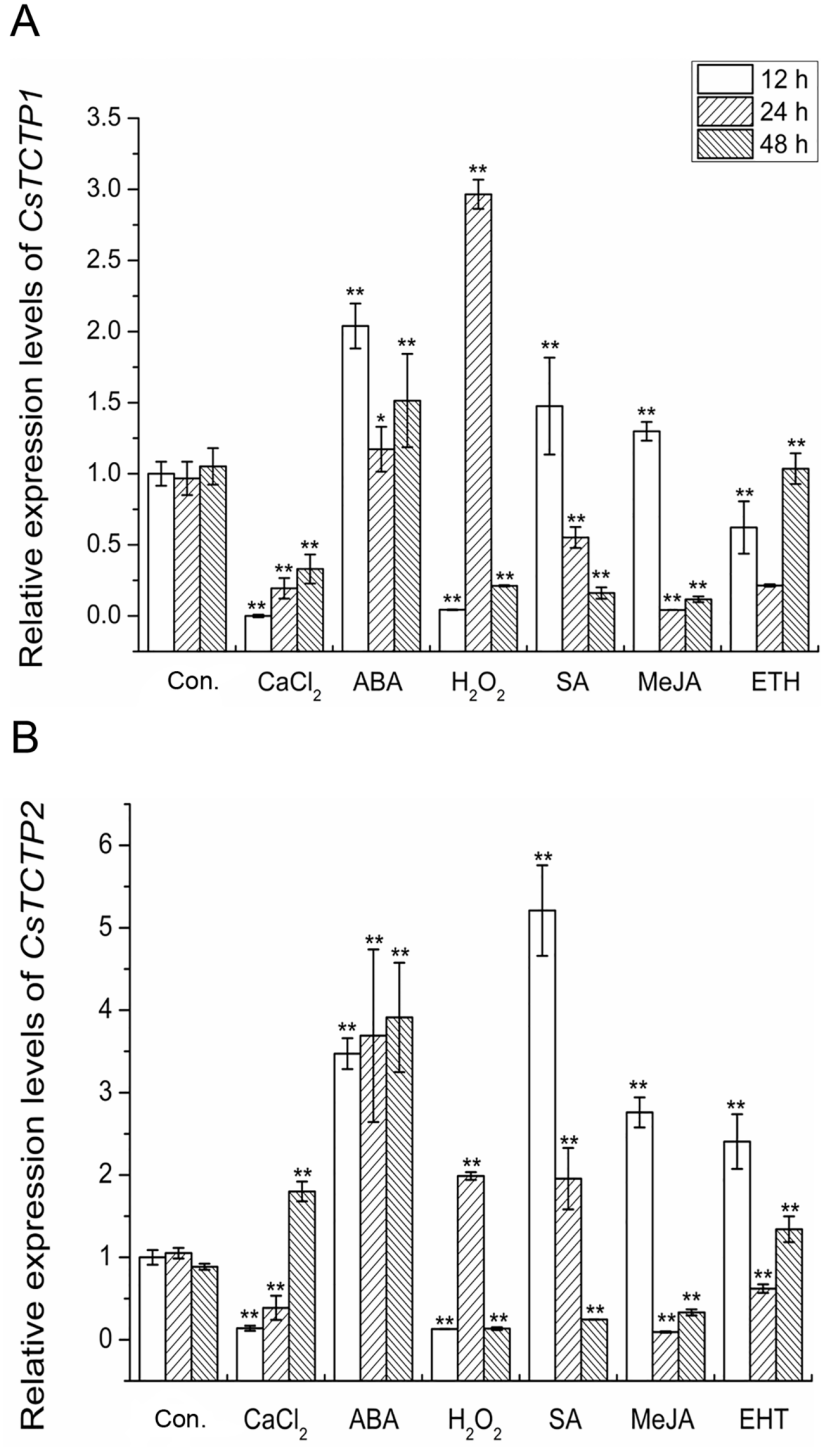
Relative expression levels of *CsTCTP1* and *CsTCTP2* in a resistant (B21-a-2-1-2) variety under various treatments. The expression level of the water treatment (12 h) was normalized as 1. Data represent means ± SEs of three biological replicates. Asterisk or asterisks indicate significant differences at P < 0.05 compared with water treatment (12 h) by Student’s *t*-test.

TCTP is also regulated by various extracellular signals [13, 17, 25]. To address whether *CsTCTP1* and *CsTCTP2* are affected by extracellular signals, we investigated their time-dependent accumulation patterns under different treatments using qRT-PCR. As shown in Fig 6, there was no visible change in *CsTCTP1* and *CsTCTP2* in leaves treated with only H_2_O. Under CaCl_2_, H_2_O_2_, ABA, MeJA, SA and Ethrel treatments, *CsTCTP1* and *CsTCTP2* showed different expression patterns. In the case of ABA treatment, *CsTCTP1* and *CsTCTP2* genes were upregulated at all time points, with the maximum upregulation observed at 12 h and 48 h post treatment, respectively. These results suggest that *CsTCTP1* and *CsTCTP2* are likely to be involved in ABA responses in cucumber plants.

### Ectopic expression of *CsTCTP1* and *CsTCTP2* in *E*. *coli* BL21 confers salt and heat tolerance

**Fig 7:**
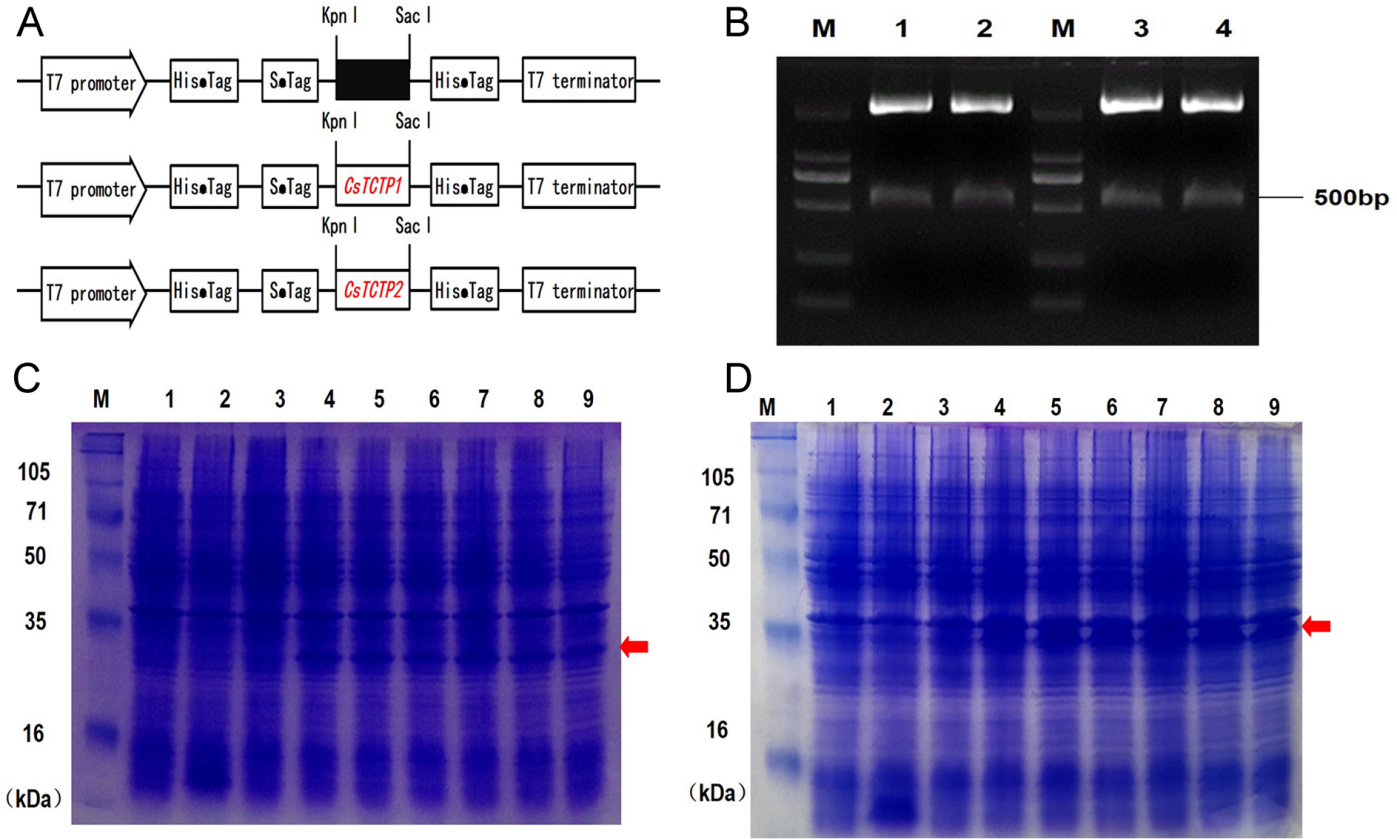
Overexpression of *CsTCTPs* in *E*. *coli*. (A) Diagram of prokaryotic expression vector construction, (B) the enzyme digestion to identify the constructed prokaryotic expression vector, M. 2000 DNA marker, 1, 2. pET30a-*CsTCTP1*/Kpn I+Sac I, 3, 4. pET30a-*CsTCTP2*/Kpn I+Sac I, (C) CsTCTP1 protein expression in *E*. *coli* BL21, M. protein marker, 1. pET30a without induction, 2. pET30a induction for 6 h, 3. pET30a-*CsTCTP1* without induction, 4, 5, 6. pET30a-*CsTCTP1* induction for 3 h, 6 h, 12 h, respectively, 7, 8, 9. Repeat 4, 5, 6, (D) CsTCTP1 protein expression in *E*. *coli* BL21, M. protein marker, 1. pET30a without induction, 2. pET30a induction for 6 h, 3. pET30a-*CsTCTP2* without induction, 4, 5, 6. pET30a-*CsTCTP2* induction for 3 h, 6 h, 12 h, respectively, 7, 8, 9. Repeat 4, 5, 6.

TCTP is involved in responses to a wide range of stimuli, such as mercury, heat, cold, drought, salt, ABA, ET and pathogens [13, 23, 26, 27]. To test whether overproduction of CsTCTP1 and CsTCTP2 can improve *in vivo* stress tolerance, *CsTCTP1* and *CsTCTP2* were cloned in pET30a and overexpressed in *E*. *coli* BL21. SDS-PAGE showed the molecular mass of CsTCTP1 to be very close to the predicted mass of 25 kDa harboring the His-tag, while CsTCTP2, although less close to the predicted mass, appeared to have the 34 kDa additional protein visualized according to results from our group (Fig 7).

**Fig 8:**
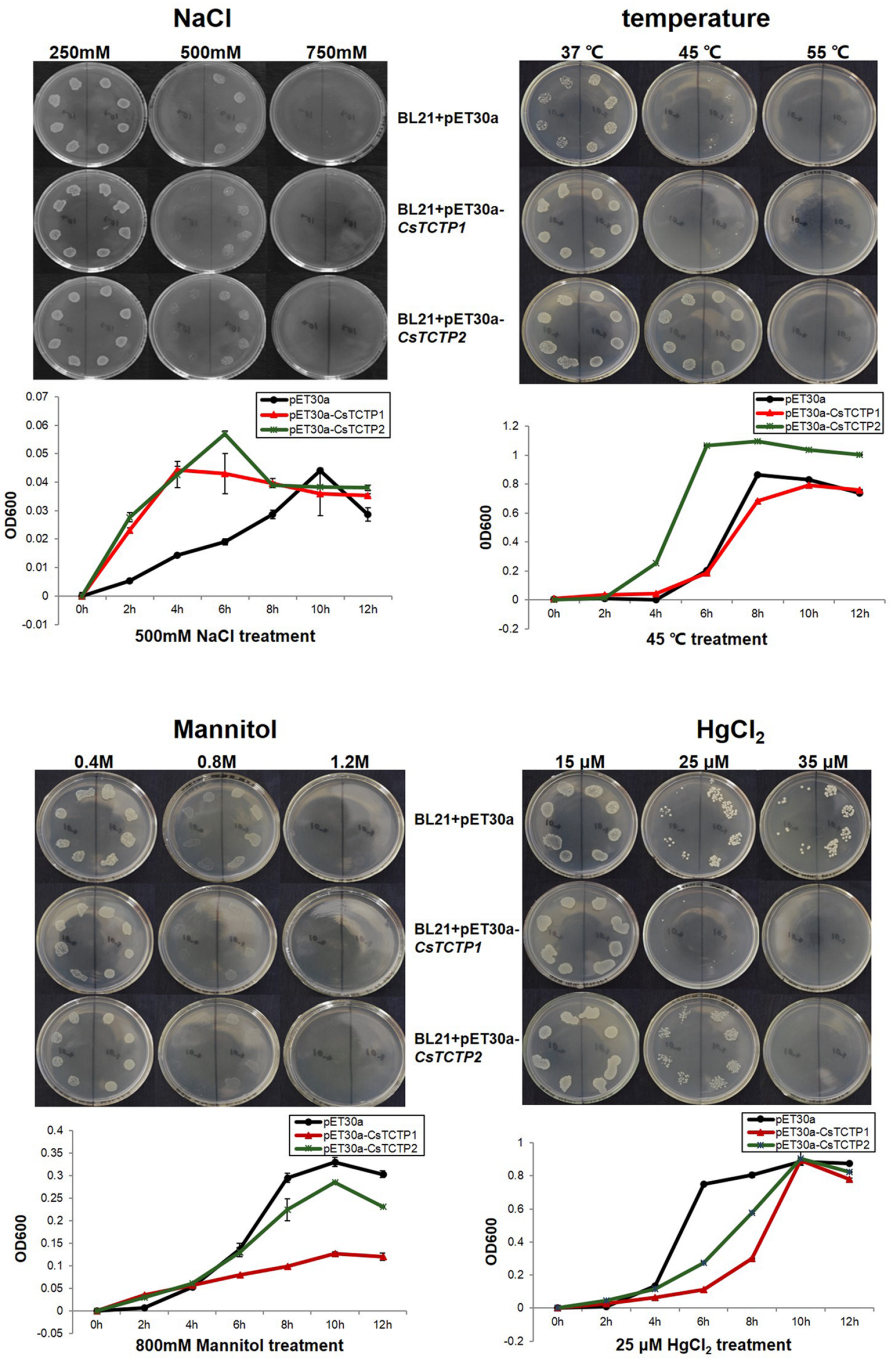
Abiotic stress assay. Spot assay of BL21+pET30a, BL21+pET30a-*CsTCTP1* and BL21+pET30a-*CsTCTP2* on LB plates containing 50 μg/mL kanamycin at 37°C, 45°C and 55°C (for heat stress) or on LB plates with NaCl (250 mM, 500 mM and 750 mM, for salt stress), mannitol (0.4 M, 0.8M and 1.2M, for drought stress), or HgCl_2_ (15 μM, 25 μM and 35 μM, for mercury stress) at 37°C overnight. Liquid culture assay on LB liquid medium containing 50 μg/mL kanamycin at 45°C or on LB liquid medium with 500 mM NaCl, 0.8M mannitol, or 25 μM HgCl_2_ at 37°C. All data points are mean ± SE (n≥3).

Control (BL21 + pET30a) and overexpressed cells (pET30a-*CsTCTP1* or pET30a-*CsTCTP2*) were exposed to salt, high temperature, drought, and HgCl_2_, and their growth was monitored (Fig 8). The results revealed that overexpressing *CsTCTP1* and *CsTCTP2* resulted in a higher tolerance to salt treatment than that of the control cells, and overexpressing *CsTCTP2* also resulted in a higher tolerance to high temperature. Furthermore, overexpressed cells and control cells showed similar growth on LB liquid medium with different supplements (Fig 8). Recombinant *CsTCTP1* and *CsTCTP2* cells had a slower growth and a lower tolerance than did the control in LB containing mannitol and HgCl_2_.

## Discussion

The high degree of homology and the common existence of TCTP in animals and plants underscore its vital roles in growth, development and responses to biotic or abiotic stresses in different organisms. However, plant TCTPs have not been studied extensively, especially in cucumber plants. In this study, the 2096 bp *CsTCTP1* promoter and 2015 bp *CsTCTP2* promoter sequences (relative to the translation start site) showed only 45% nucleotide identity. By contrast, the *CsTCTP1* and *CsTCTP2* coding sequences share 73% identity. Indeed, *CsTCTP1* and *CsTCTP2* displayed slightly different expression patterns. A putative *cis*-element associated with defense and stress responses (TC-rich repeats) was identified in the *CsTCTP1* and *CsTCTP2* promoter sequences. Similarly, the *CsTCTP2* promoter sequence contains Box-W1, which is related to fungal elicitor responses. These data are in line with the expression data showing that *CsTCTP1* and *CsTCTP2* genes are affected by abiotic and biotic stresses. In addition, *CsTCTP1* and *CsTCTP2* slightly differed in their evolution and structures. It is reasonable to assume that such differences are reflected in their functions.

In *Drosophila*, dTCTP as a GEF directly associates with dRheb GTPases and positively regulates the TOR signaling pathway [4, 28]. TOR signaling pathway is known as a central coordinator of nutrient, energy and stress signaling networks [29]. In *Arabidopsis*, AtTCTP can bind to four AtRab GTPases (AtRABA4a, AtRABA4b, AtRABF1 and AtRABF2b) and interact with *Drosophila* dRheb GTPases. Similarly, dTCTP can bind to the four *Arabidopsis* AtRab GTPases [9]. Furthermore, eukaryotic GTPases act as molecular switches for diverse cellular processes. This GTPase-binding property might explain how TCTP is a multi-functional protein [17, 30]. *In silico* analysis of CsTCTP1 and CsTCTP2 functional domains indicated that both share the key GTPase binding surface. Although the GEF property of CsTCTP has not been shown *in vitro*, it is clear that CsTCTP is an important component of the TOR pathway.

Plant developed a range of defense mechanisms to protect themselves from external environmental stimuli. The expression of stress-responsive genes is an important part of the plant response to a variety of biotic and abiotic stresses. In plants, the accumulation of the *TCTP* gene by fungal stress has been reported in Arabidopsis (*Pseudomonas syringae*) and wheat (*Erysiphe graminis*) [24, 31]. CsTCTP1 was identified in *S*. *fuliginea*-resistant cucumber cultivar interactions [12]. In contrast with the study conducted by Zheng [32], in the current study, the pathogen activated the early accumulation of *CsTCTP1* and *CsTCTP2*. In this study, the maximum accumulation of *CsTCTP1* and *CsTCTP2* in the resistant variety B21-a-2-1-2 appeared earlier than that in the susceptible variety upon inoculation with *S*. *fuliginea*. From these results, it is clear that *CsTCTP1* and *CsTCTP2* both function at early stages (24 h and 48 h post infection) in plant resistance to pathogen attack. *CsTCTP1* and *CsTCTP2* levels are also highly regulated in response to abiotic stresses, such as heat, cold, drought and salinity stress. These results are in accordance with the *in silico* analysis data showing that *CsTCTP1* and *CsTCTP2* genes are affected by various abiotic stresses. As *CsTCTP1* and *CsTCTP2* genes were found to be upregulated at all treatment time points, this ABA-regulating property might explain the involvement of CsTCTP in the seemingly related ABA signaling transduction process. The results of promoter analysis and qRT-PCR showed that *CsTCTP1* and *CsTCTP2* might have diverse functions in a variety of stress responses and ABA signaling transduction processes in cucumber plants. Therefore, *CsTCTP1* and *CsTCTP2* might be good candidates for alternative stress-related genes.

Several stress-related *cis*-elements were identified in the promoters of *CsTCTP1* and *CsTCTP2*, as supported by their rapid induction under various stresses. However, because of these results, it is not clear whether *CsTCTP1* and *CsTCTP2* are positively regulated or negatively regulated in response to various abiotic stresses. Studies on the specific function of *CsTCTP1* or *CsTCTP2* in plant-stress interactions are necessary. Current studies indicate that overproduction of plant stress-related genes enhances growth of *E*. *coli* cells [19, 33]. Furthermore, a cassava TCTP conferred salt stress tolerance to *E*. *coli* [21]. The fact that the pET30a-*CsTCTP2* sample has a predicted protein band of 26 kDa was inconsistent with the additional protein band of about 34 kDa that was not found in the control sample. One explanation of the results is as follows: (a) adding SDS changed the protein conformation (b) the terminator of CsTCTP2 may not terminate translation effectively until it meets the terminate of the vector. Mercury (Hg) is also a growth risk factor to plants that damages many cellular-level functions and inhibits plant growth and development. It has been shown that *OsTCTP* plays an important role in mercury tolerance in rice [22]. Intriguingly, overexpression of *CsTCTP1* and *CsTCTP2* reduced the tolerance to HgCl_2_ stress. Overproduction of CsTCTP1 and CsTCTP2 resulted in greater sensitivity to drought and HgCl_2_ stresses. Collectively, CsTCTP plays an important role in cucumber abiotic stress responses. Since the response mechanisms of CsTCTP to different stresses may differ, further study of CsTCTP is necessary.

In conclusion, *CsTCTP1* and *CsTCTP2* promoter, full-length DNA and genomic sequences were cloned from *Cucumis sativus*. *CsTCTP* might be involved in stress responses by regulating ABA or TOR pathways in cucumber. *CsTCTP* is related to responses to a wide range of stimuli. *CsTCTP1* and *CsTCTP2* showed positive responses to salt and heat stresses and negative responses to drought and HgCl_2_ stresses.

## Supporting information

S1 Table Predicted *cis*-acting elements with putative functions identified in the *CsTCTP1* promoter using the PLACE and PlantCARE databases. (RTF)

S2 Table Predicted *cis*-acting elements with putative functions identified in the *CsTCTP2* promoter using the PLACE and PlantCARE databases. (RTF)

S3 Table List of primers used in the study. (RTF)
