# Supplementary material for: Molecular characterization, expression analysis and heterologous expression of two translationally controlled tumor protein genes from Cucumis sativus
Source: PLoS One. 2017 Sep 19;12(9):e0184872. doi: 10.1371/journal.pone.0184872 (PMC5605047; doi:10.1371/journal.pone.0184872)
Supplement: S1 Table — (RTF) [file pone.0184872.s001.rtf]

Supporting information	 
S1 Table. Predicted cis-acting elements with putative functions identified in the CsTCTP1 promoter using PLACE and PlantCARE databases
Site name	Location	Sequence	Function	
ABRE	112(-),524(+),293(-)	TACGTG	involved in abscisic acid responsiveness	
ACE	319(-)	CTAACGTATT	involved in light responsiveness	
BoxⅠ	489(-)	TTTCAAA	light responsive element	
G-Box	112(+),524(-),293(+)	CACGTA	involved in light responsiveness	
G-Box	112(-),524(+),293(-),956(+)	TACGTG	involved in light responsiveness	
GA-motif	1131(+),1289(+)	ATAGATAA	part of a light responsive element	
GAG-motif	1437(-)	AGAGAGT	part of a light responsive element	
GT1-motif	661(-),1364(-),662(-)	GGTTAAT	light responsive element	
HSE	1045(+)	AAAAAATTTC	involved in heat stress responsiveness	
MBS	910(-)	CAACTG	involved in drought-inducibility	
Sp1	1375(+),1449(+),1448(+)	CC(G/A)CCC	light responsive element	
TC-rich repeats	938(-),1042(-),964(-)	ATTTTCTTCA	involved in defense and stress responsiveness	
TCA-element	1287(-)	CCATCTTTTT	in salicylic acid responsiveness	
TCT-motif	57(+),670(+)	TCTTAC	part of a light responsive element	
TCT-motif	381(-)	CAANNNNATC	involved in circadian control	
