# Supplementary material for: Molecular characterization, expression analysis and heterologous expression of two translationally controlled tumor protein genes from Cucumis sativus
Source: PLoS One. 2017 Sep 19;12(9):e0184872. doi: 10.1371/journal.pone.0184872 (PMC5605047; doi:10.1371/journal.pone.0184872)
Supplement: S2 Table — (RTF) [file pone.0184872.s002.rtf]

Supporting information	 
S2 Table. Predicted cis-acting elements with putative functions identified in the CsTCTP2 promoter using PLACE and PlantCARE database
Site name	Location	Sequence	Function	
3-AF3 binding site	618(+)	CACTATCTAAC	part of a conserved DNA module array	
AAAC-motif	631(+)	CAATCAAAACCT	light responsive element	
ABRE	1138(-)	CACGTG	involved in abscisic acid responsiveness	
ACE	299(-)	AAAACGTTTA	involved in light responsiveness	
AE-box	158(+)	AGAAACAA	part of a module for light response	
ARE	691(-)	TGGTTT	essential for the anaerobic induction	
Box 4	484(+),1216(-),591(+),1220(-)	ATTAAT	involved in light responsiveness	
BoxⅠ	1036(-)	TTTCAAA	light responsive element	
Box Ⅲ	773(+)	CATTTACACT	protein binding site	
Box-W1	821(-)	TTGACC	fungal elicitor responsive element	
CATT-motif	212(-)	GCATTC	part of a light responsive element	
ELI-box3	691(+)	AAACCAATT	elicitor- responsive element	
ERE	1305(-)	ATTTCAAA	ethylene- responsive element	
G-Box	1000(-),1138(-)	CACGTT	involved in light responsiveness	
GA-motif	988(+),1441(-)	ATAGATAA	part of a light responsive element	
GAG-motif	604(-)	GGAGATG	part of a light responsive element	
GCN4_motif	1054(-)	TGAGTCA	involved in endosperm expression	
I-box	42(-)	ATGATATGA	part of a light responsive element	
MRE	674(-)	AACCTAA	MYB binding site involved in light responsiveness	
O2-site	42(-),	GATGACATGA	involved in zein metabolism regulation	
Skn-1_motif	37(+),238(-)	GTCAT	required for endosperm expression	
TC-rich repeats	697(+),1346(-),1180(+),1461(+)	ATTTTCTTCA	involved in defense and stress responsiveness	
TCT-motif	227(-),272(+)	TCTTAC	part of a light responsive element	
as-2-box	48(-)	GATAATGATG	involved in shoot-specific expression and light responsiveness	
chs-CMA1a	1225(-)	TTACTTAA	part of a light responsive element	
circadian	361(+)	CAANNNNATC	involved in circadian control	
