# Supplementary material for: Molecular characterization, expression analysis and heterologous expression of two translationally controlled tumor protein genes from Cucumis sativus
Source: PLoS One. 2017 Sep 19;12(9):e0184872. doi: 10.1371/journal.pone.0184872 (PMC5605047; doi:10.1371/journal.pone.0184872)
Supplement: S3 Table — (RTF) [file pone.0184872.s003.rtf]

Supporting information
S3 Table. List of primers used in the study.
Name	Primer	
1-cDNA	F: 5'-CGGGGTACCCCGTTGGTTTATCAGGAC-3' 
R: 5'-CGAGCTCGTCAGCACTTGACTTCCTTCAAAC-3'	
2-cDNA	F: 5'-CGGGGTACCCCGCTTCTCTACCAAGACCTT-3'
R: 5'-CGAGCTCGTCAACACTTGACTTCCTTCAACC-3'	
1-DNA	F: 5'-TACGAAGTTCTTAACCAAAACCCTCC-3'
 R: 5'-CGGGATCCCGTTGTTGATATTATAAAATAGAG-3'	
2-DNA	F: 5'-CGAGCTCGAATCTTCCTTCTTCTTTGAA-3' 
R:5'-CGGGATCCCGTTGACAAATTTTTAATCTCT-3'	
1-P	F: 5'-GGTTGAGAAAAGAAAGTTAGGACCGTACC-3'
R: 5'-CGGGATCCCGTAATTCAATCGAATATCAC-3'	
2-P	F: 5'-CGAGCTCGAAGAATGTCTCACACCTAA-3' 
R: 5'-GGCTGTGGCTGCAAAAGAGAATTGAAAAA-3'	
1-RT	F: 5'-GGACAAGAAGGTTTTCCTCACA-3'
R: 5'-GGAACTTAACTGCTCCCTCAAT-3'	
2-RT	F: 5'-AGACGAAGAAAAGCAAGAGTTG-3'
R: 5'-TCCCTTCACCCACAAAGAAT-3'	
18SrRNA	F: 5'-ATGATAACTCGACGGATCGC-3'
R: 5'-CTTGGATGTGGTAGCCGT-3'	
